# Evaluation of ultrasound accuracy in thyroid mass measurement and its impact on ^131^I treatment for Graves’ disease

**DOI:** 10.3389/fendo.2025.1617229

**Published:** 2025-07-25

**Authors:** Xiangxiang Li, Xu Han, Nan Liu, Shen Wang, Hongyuan Zheng, Ziyu Ma, Ruiguo Zhang, Qiang Jia, Wei Zheng

**Affiliations:** Department of Nuclear Medicine, Tianjin Medical University General Hospital, Tianjin, China

**Keywords:** Graves’ disease, thyroid mass, ^131^I treatment efficacy, clinical predictive model, CT calibration

## Abstract

**Background:**

Thyroid mass is crucial for ^131^I treatment of Graves’ disease (GD). However, the accuracy of ultrasound (US) - based thyroid mass measurement remains controversial.

**Methods:**

This retrospective study included patients who underwent thyroid US and CT scans. The differences correlation, and agreement in thyroid mass measurements between the two methods were analyzed. Data from GD patients who received their first ^131^I treatment were collected and evaluated at a 6-month follow-up. Regression analyses identified clinical factors for treatment efficacy and developed a predictive model.

**Results:**

A statistically significant difference was observed in thyroid mass measurements exceeding 20 g between US and CT. (Z = -11.493, P<0.001). Despite a strong correlation between the two methods (r = 0.9809, P=0.001), the average relative error remained substantial (0.19 ± 11.65%). Poor agreement was observed between CT and US (mean bias: 16.65g; ICC = 0.179, p = 0.087). Disease duration, FT_4_ level, 24 - hour radioactive iodine uptake, ^131^I dose and thyroid mass were identified as independent risk factors influencing the efficacy of the initial ^131^I treatment (p<0.05). Based on these factors, a predictive model was developed and evaluated using ROC curves, DCA and CAL. The model demonstrated an AUC of 0.663 (95% CI = 0.631-0.695).

**Conclusion:**

US may underestimate the true thyroid mass in large-mass cases; therefore, CT calibration is recommended before initiating ^131^I treatment. The proposed predictive model provides valuable guidance for optimizing initial ^131^I treatment in patients with GD.

## 1 Background

Hyperthyroidism is a clinical syndrome characterized by excessive thyroid hormones levels in the bloodstream due to various causes (1). Graves’ disease (GD) is the most common etiology of hyperthyroidism (2). Treatment options for GD include iodine-131 (^131^I), antithyroid drugs (ATD) and surgery (3). Although ATDs are the first-line treatment, relapse occurs in approximately 50% of patients following treatment discontinuation (4). ^131^I treatment is widely favored by clinicians due to its well-established safety profile, particularly in cases where ATDs are contraindicated or when patients fail to achieve euthyroidism with ATD therapy (5).

Studies have shown that multiple factors influence the efficacy of ^131^I treatment in GD, including gender, age, prior use of ATDs, free thyroxine (FT_4_) levels, thyroid mass, and the ^131^I dose (6). Among these, thyroid mass is a crucial parameter in determining the appropriate ^131^I dose for GD patients (7). Multiple studies have emphasized its influence on the treatment response of GD patients (8–10). Currently, thyroid mass estimation methods include palpation, ultrasound (US), computer tomography (CT), and radionuclide imaging, with US being the most commonly used in clinical practice. The standard US measurement follows the ellipsoid volume formula, V = π/6 × L × W × T. However, a cadaveric study (11) suggested that adjusting the correction factor from 0.524 to 0.479 could improve measurement accuracy. A multi-method thyroid measurement study (12) indicates that compared with CT measurements, US estimates are on average 20.06 ± 8.31 g smaller than CT methods. Research (13) has also highlighted significant discrepancies between thyroid volumes measured by US and those determined intraoperatively, with measurement errors increasing as thyroid volume enlarges. In contrast, CT is a well-established technique for thyroid volume assessment, offering a higher degree of accuracy (14). One phantom study demonstrated exceptional agreement between CT-measured and actual thyroid volumes, with a mean error of just 0.27 ± 1.53% (15). The aim of this study is to evaluate the accuracy of US in estimating thyroid mass, using CT as the reference standard and to identify the critical threshold for significant differences between the two methods. To visualize the differences between CT and US thyroid mass measurements, Sankey diagrams were used to display the distribution patterns across both methods. The findings will enable nuclear medicine practitioners to more accurately prescribe ¹³¹I dosages for GD treatment, better understand the impact of thyroid mass on ¹³¹I treatment efficacy, and develop a prognostic model for predicting treatment outcomes.

## 2 Materials and methods

### 2.1 Enrollment of patients

The clinical data of 192 patients who underwent both thyroid US and SPECT/CT examinations in the Nuclear Medicine Department of Tianjin Medical University General Hospital between October 2022 and December 2024 were retrospectively recruited to compare the differences in thyroid mass measurements between US and CT. Patients with congenital thyroid malformations, a history of thyroid surgery, or those who were pregnant were excluded. Additionally, data from 1,584 patients with GD were included to investigate the impact of thyroid mass on the initial ^131^I treatment for GD. These patients underwent thyroid US in the same department between June 2022 to June 2024. The inclusion criteria were as follows: (i) Diagnosis of GD followed the guidelines of the Chinese Society of Nuclear Medicine (2021) (16); (ii) No contraindications to radioactive iodine and undergoing initial ^131^I treatment; (iii) Follow-up period of at least 6 months. The exclusion criteria included: (i) history of thyroid surgery; (ii) Incomplete or missing clinical data, or loss to follow-up; (iii) Presence of other malignant conditions; (iv) Pregnancy or lactation. The study was approved by the Ethics Committee of Tianjin Medical University General Hospital (Approval numbers: IRB2025-YX-111-01).

### 2.2 Clinical data collection

Clinical information was collected for each patient, including gender, age, duration of hyperthyroidism, history of ATDs administration, levels of Free Triiodothyronine (FT_3_), Free Thyroxine (FT_4_), Thyroid Stimulating Hormone (TSH), Thyroglobulin Antibody (TgAb), Thyroid Peroxidase Antibody (TPOAb), TSH Receptor Antibody (TRAb), 24-hour Radioactive Iodine Uptake (24h RAIU), thyroid gland mass, the effective half - life (T1/2e), and the ^131^I dose administered. All patients were provided with detailed explanations of the procedure and necessary precautions, which included maintaining a low-iodine diet and avoiding iodide containing medications for 7–14 days prior to treatment. ATDs were required to be discontinued at least 3 days before ^131^I treatment. Personalized dose of ^131^I were calculated as follows (17): ^131^I (mCi) = $\frac{0.67\times \;absorption\;dose\;\left(Gy/g\right)\;\times \;estimated\;thyroid\;mass\;\left(g\right)}{the\;maximum\;RAIU\;\left(\%\right)\;\times \;T1/2e\;\left(d\right).}$ . The absorption dose is set at 110 Gy/g.

### 2.3 Thyroid mass measurement

Standardized thyroid ultrasound measurements were performed as follows: Patients were positioned supine with neck hyperextension. The thyroid gland dimensions were measured using a Mindray Resona 8 color Doppler US scanner with a 10 MHz high-frequency linear probe by a trained sonographer. All measurements obtained at end-expiration breath-hold. The volume (V) of each lobe was calculated using the formula (11): V = 0.479 × length × width × thickness (cm3). The total thyroid volume was determined by summing the volumes of both lobes, and the thyroid mass (g) was calculated based on a specific gravity of 1.0. For CT imaging, a CZT-SPECT/CT (Discovery NM/CT 670 CZT; GE Healthcare) equipped with a wide-energy high-resolution collimator was used for acquisition. CT was performed for GD patients who had undergone US, with the interval between the two examinations no more than three days. The CT scanning parameters were as follows: tube voltage of 140 kV, tube current of 220 mA, slice thickness of 2.5 mm, and matrix size of 512 × 512. For the 192 eligible patients, thyroid mass measurements obtained by US were categorized into 5 groups (≤20, 21-40, 41-60, 61-80, >80 g) to facilitate comparative analysis of differences and correlations between the two methods. Additionally, for a more detailed visualization of measurement distribution and inter-method variability, the data were further stratified into 10 cohorts (≤10, 11-20,…, >90 g). A Sankey diagram was employed to visualize the flow patterns and differences between the two measurement approaches.

### 2.4 Efficacy evaluation

Serum thyroid function indices were measured in GD patients 6 months after ^131^I treatment to assess treatment efficacy. Therapeutic efficacy was evaluated using the following criteria (16). Complete remission: Complete resolution of hyperthyroidism symptoms and signs, with FT_4_ levels returning to normal. Hypothyroidism: Onset of hypothyroidism symptoms and signs, with FT_3_ and FT_4_ levels below normal and TSH levels above average. Partial remission: Alleviation of hyperthyroidism symptoms, with a reduction in FT_4_ levels, though not returning to normal. Inefficacy: No improvement in symptoms, with possible aggravation and no significant change in FT_4_ levels. Both complete remission and hypothyroidism were classified as “cure” (cured group), while partial remission and inefficacy were classified as “uncured” (uncured group).

### 2.5 Statistical analysis

We used SPSS 26.0 for statistical analysis of the data. For continuous variables with non - normal distribution, the median and inter - quartile span were utilized. The Mann-Whitney test was applied to compare differences between two groups of such data. Chi-square was performed for categorical data analysis. Inter-method agreement in thyroid mass measurements was evaluated using both intraclass correlation coefficient (ICC) and Bland-Altman plots. Logistic regression analysis was carried out with variables that showed statistical significance for the outcome. A p value of less than 0.05 was considered statistically significant. The receiver operating characteristic (ROC) curve, calibration curve (CAL), decision curve (DCA) and nomogram model were obtained using R software package (4.1.3).

## 3 Results

### 3.1 Comparison of thyroid mass measurements between CT and US

**Figure 1:**
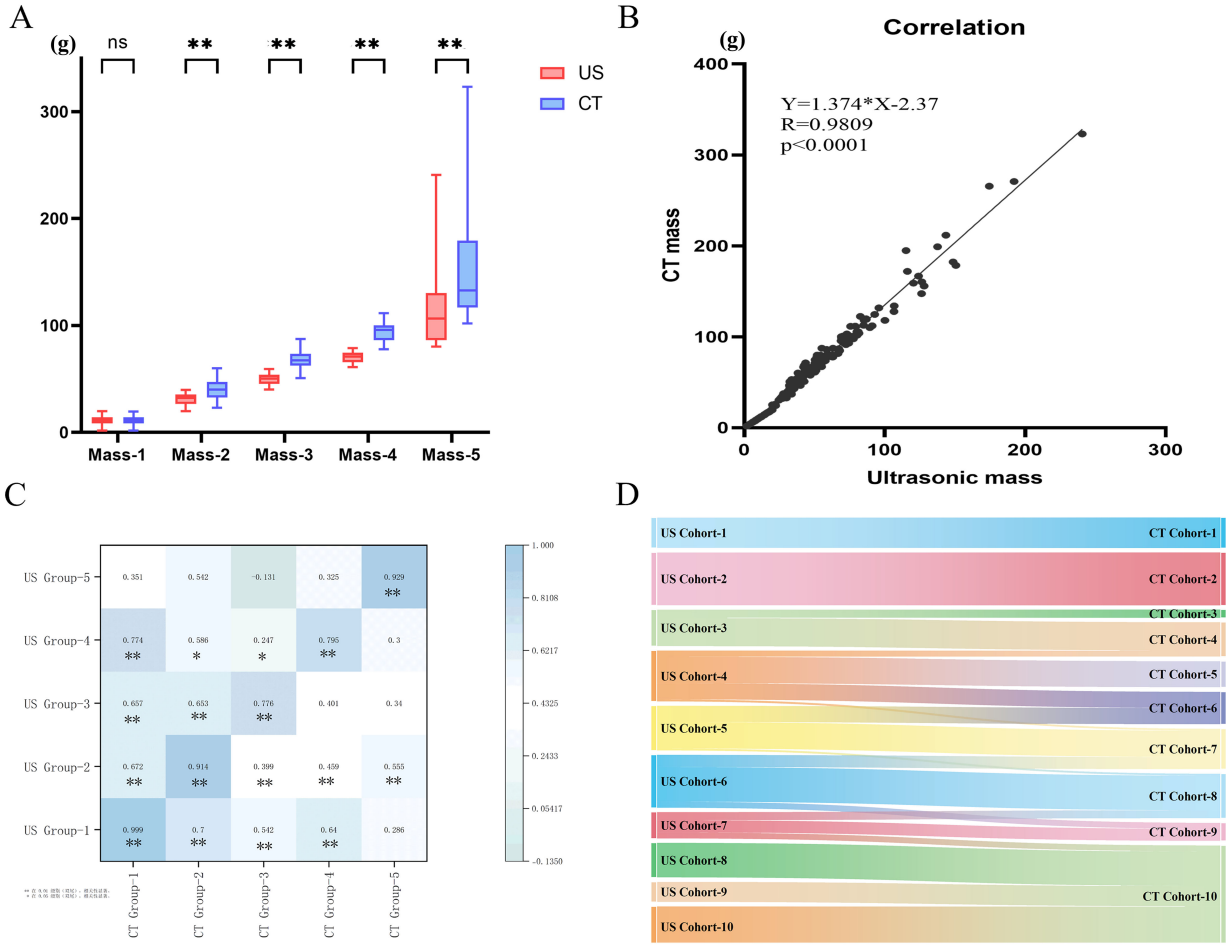
**(A)** Comparison of thyroid mass measured by US and CT; **(B)** Correlation analysis of thyroid mass measured by US and CT; **(C)** Correlation Among five Groups; **(D)** The flow direction between CT and US. ns: p > 0.05; *: p < 0.05; **: p < 0.01.

**Figure 2:**
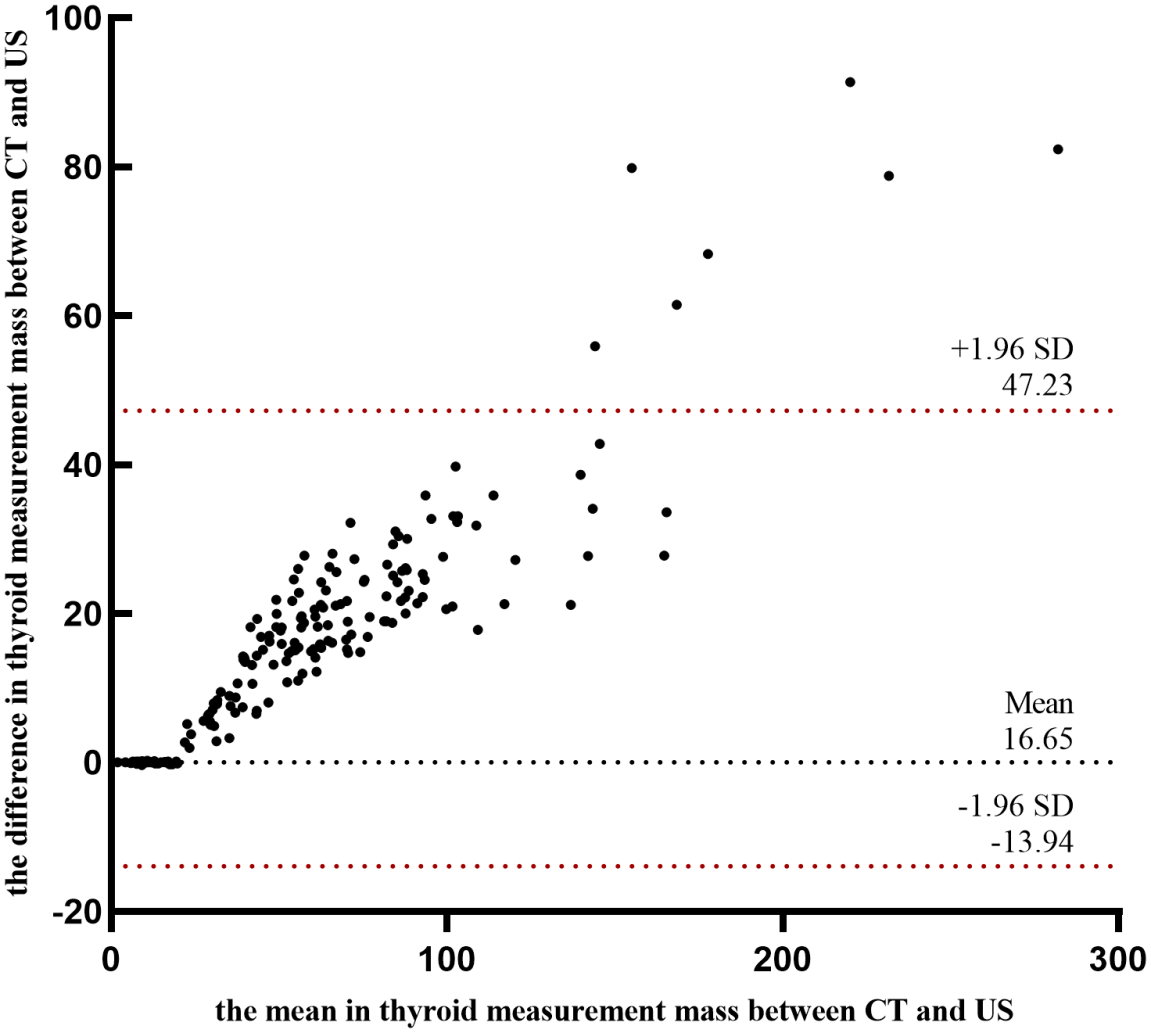
Bland-Altman plot of CT and US thyroid mass measurements.

A comparison of thyroid gland mass measurements between CT and US demonstrated a statistically significant difference (Z = -11.493, P < 0.001). However, in pairwise group comparisons, no significant difference was observed between CT and US in Group 1 (Z = -0.628, P = 0.530), whereas significant differences were detected in all other groups (**Table 1**, **Figure 1A**). The mean relative error between US and CT measurements was 0.19 ± 11.65%. Despite this, a strong correlation was observed between the two methods (r = 0.981, P < 0.001) (**Figure 1B**). Further subgroup analysis also demonstrated strong correlations between CT and US measurements across all groups (**Figure 1C**). And we employed Sankey diagrams to visually demonstrate the flow distribution and measurement discrepancies of thyroid mass between the two methods. In the US group, the majority of patients were in cohorts 2 (10–20 g), 4 (30–40 g), 5 (40–50 g), and 6 (50–60 g). In contrast, in the CT group, the predominant cohorts were 2 (10–20 g), 7 (60–70 g), 8 (70–80 g), and 10 (> 90 g). Furthermore, as shown in **Figure 1D**, as thyroid mass increases, US-based cohorts tend to correspond to higher CT-based groups, indicating an increasing margin of error in US measurements for larger thyroid glands. The agreement between the two methods for thyroid mass measurements showed an ICC of 0.179 (p=0.087). Bland-Altman analysis revealed a systematic bias of (16.65 ± 15.60g), with CT measurements consistently higher than US values (**Figure 2**). This suggests that the US may underestimate the actual thyroid mass in such cases. Therefore, CT calibration is recommended for thyroid glands exceeding 20 g to improve measurement accuracy.

**Table 1:** Comparison of thyroid volume measurement by US and CT.

Groups	US	CT	Z	P
Group-1 (n=41)	9.73 (7.61,12.79)	9.75 (7.65,12.92)	-0.628	0.53
Group-2 (n=43)	27.73 (25.94,34.22)	35.40 (32.16,45.57)	-5.711	<0.001
Group-3 (n=48)	46.67 (42.84,50.24)	65.24 (59.65,70.34)	-6.031	<0.001
Group-4 (n=30)	71.07 (65.88,74.70)	95.98 (86.28,95.99)	-4.782	<0.001
Group-5 (n=30)	106.61 (86.36,130.45)	132.85 (116.68,179.43)	-4.783	<0.001

### 3.2 Treatment outcome

Among the 1,584 patients undergoing initial ¹³¹I treatment, the median disease duration was 24 months (IQR, 12 to 84). The majority were female, accounting for 74.3% (1,175/1,584) of the cohort. Before treatment, thyroid mass ranged from 4 to 302.9 g, with a median of 28.3g (IQR, 19.93 to 45.10). Patients’ ages ranged from 11 to 84 years, with a mean age of 42.58 ± 14.53 years. Additionally, a significant proportion of 72.35% (1,146/1,584) had a history of prior ATD therapy. Patients received ¹³¹I doses ranging from 2 to 30 mCi. The overall cure rate for GD patients treated with ¹³¹I was 70.77% (1,121/1,584). A comparison of cure rates among different mass groups is presented in **Table 2**. Group 1 achieved the highest cure rate at 81.6% (319/391), whereas Group 5 had the lowest at 50% (56/112). This trend suggests that as thyroid mass increases, the cure rate progressively decreases.

**Table 2:** Cure rates six months after ^131^I treatment in GD patients with different thyroid masses.

Thyroid mass	Total	Cure rate (%)	Uncured rate (%)	χ2	P
Group-1	391	319 (81.6)	72 (18.4)	68.496	<0.001
Group-2	679	501 (73.8)	178 (26.2)		
Group-3	281	176 (62.6)	105 (37.4)		
Group-4	121	69 (57.0)	51 (43.0)		
Group-5	112	56 (50.0)	56 (50.0)		
Total	1584	1121	463		

### 3.3 Establishment of clinical prediction mode

A univariable analysis was conducted to assess potential factors influencing treatment outcomes. The results demonstrated statistically significant differences in remission rates 6 months after ¹³¹I treatment across disease duration, FT_3_, FT_4_, 24h RAIU, ^131^I dosage, and thyroid mass (all P<0.05). Subsequently multivariate logistic regression analysis indicated disease duration (OR = 1.002, 95% CI = 1.000 - 1.004, p = 0.016), FT_4_ (OR = 1.01, 95% CI = 1.01 - 1.02, P<0.001), 24h RAIU (OR = 0.99, 95% CI = 0.98 - 0.99, P = 0.001), thyroid mass (OR = 1.008, 95% CI = 1.004 - 1.013, P<0.001), and ^131^I dose (OR = 1.07, 95% CI = 1.05 - 1.10, P<0.001) as key factors influencing ^131^I treatment efficacy in GD (**Table 3**).

**Table 3:** Univariate and multivariate logistic regression analysis of factors influencing therapeutic effect after ^131^I treatment in GD patients.

Variables	Single factor		Multiple factor	
	OR (95%CI)	P	OR (95%CI)	P
The use of ATD				
0	1.00 (Reference)			
1	1.21 (0.94 ~ 1.54)	0.138		
Disease course	1.01 (1.00 ~ 1.012)	0.001**	1.002 (1.00 ~ 1.004)	0.016*
FT_3_	1.02 (1.01 ~ 1.03)	<.001**	1.00 (0.98 ~ 1.01)	0.609
FT_4_	1.01 (1.01 ~ 1.02)	<.001**	1.02 (1.01 ~ 1.03)	<.001**
TSH	1.03 (0.63 ~ 1.70)	0.904		
TGAb	1.00 (0.98 ~ 1.02)	0.631		
TRAb	1.01 (0.99 ~ 1.03)	0.099		
TPOAb	1.00 (0.99 ~ 1.01)	0.174		
24h-RAIU	0.99 (0.98 ~ 0.99)	0.026*	0.99 (0.98 ~ 0.99)	0.001**
^131^I dose	1.09 (1.06 ~ 1.11)	<.001**	1.07 (1.05 ~ 1.10)	<.001**
Thyroid mass	1.02 (1.01 ~ 1.02)	<.001**	1.008 (1.004 ~ 1.013)	<.001**
Age	1.00 (0.99 ~ 1.00)	0.667		

OR, Odds Ratio; 95% CI, Confidence interval. *: P<0.05; **: P<0.01.

Significant predictors identified through multivariate regression analysis were used to construct ROC curves to predict the efficacy of ¹³¹I treatment in patients with GD. When thyroid mass alone was used as a predictive factor, the area under the curve (AUC) was 0.631 (95% CI = 0.595 - 0.657, P <0.001). Based on the Youden index, the optimal cut-off value for thyroid mass was determined to be 35.6 g. Patients with a mass < 35.6g achieved a cure rate of 77.7% (765/985), whereas those with a mass ≥ 35.6g had a significantly lower cure rate of 59.4% (356/599). The difference between the two groups was statistically significant (P < 0.01). These findings suggest that in patients with thyroid masses ≥ 35.6 g, conventional ¹³¹I treatment may be insufficient to achieve clinical remission.

**Figure 3:**
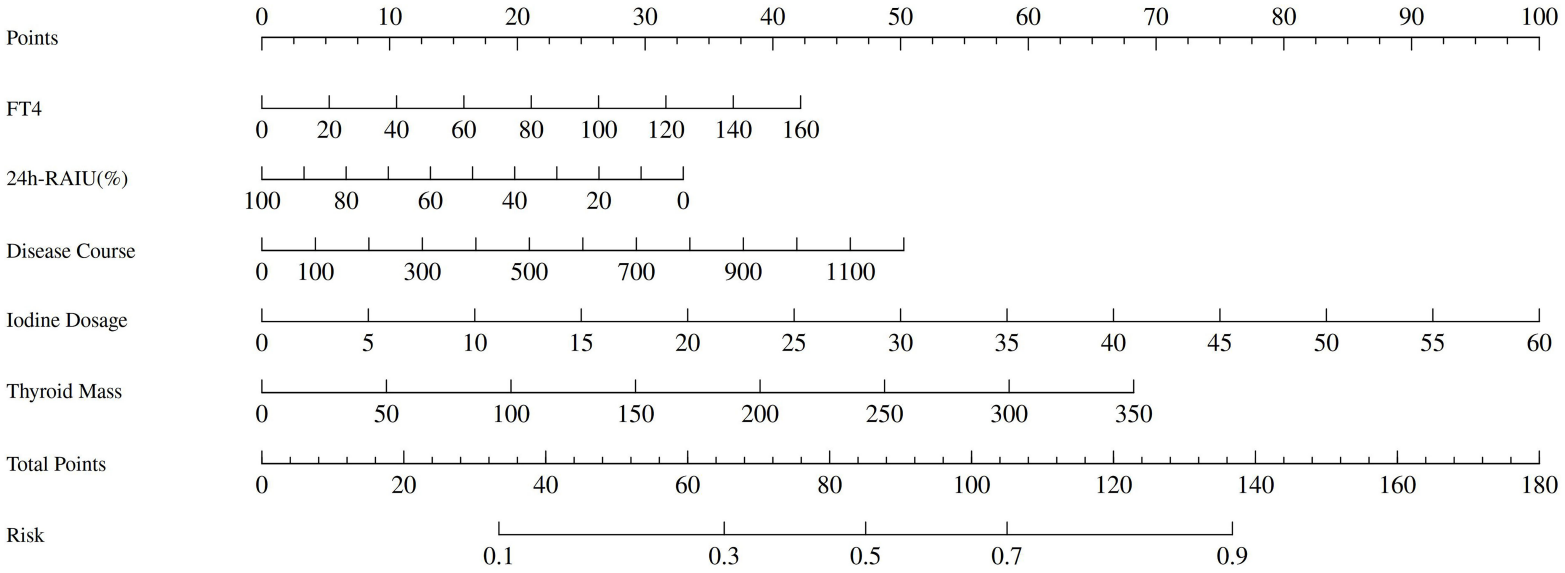
Nomogram model plot of 6-month efficacy of initial ^131^I treatment of GD patients.

**Figure 4:**
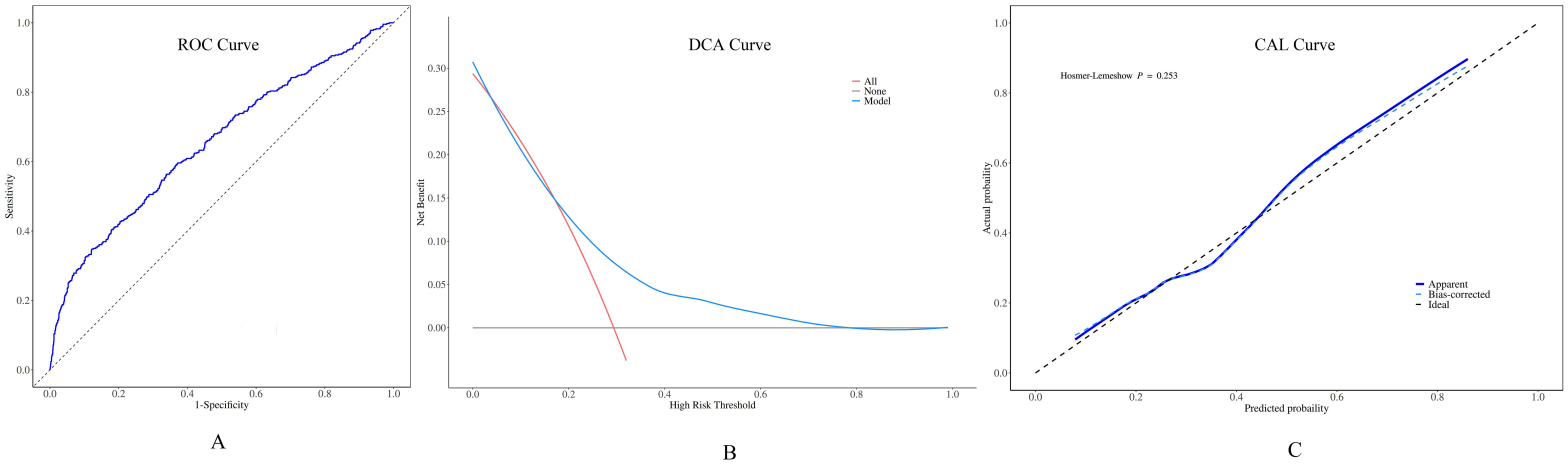
Performance of the preoperative predictive model for ¹³¹I treatment for GD. **(A)** Receiver operating characteristic (ROC) curves: the area under the curve (AUC) value was 0.663 (95% CI = 0.631 - 0.695). **(B)** When the decision curve analysis (DCA) shows that the risk threshold was greater than 0.2, this model was capable of offering a positive net benefit. **(C)** The calibration curve (CAL) manifested a high - level agreement between the predicted values from the model and the actual values.

A Nomogram model constructed using disease duration, FT_4_, 24h RAIU, ^131^I administration dose, and thyroid mass as prognostic factors is presented in **Figure 3**. The model demonstrated moderate predictive performance for the efficacy of initial ^131^I treatment in GD patients, with AUC of 0.663 (95% CI = 0.631 - 0.695, p < 0.001), a sensitivity of 44.4%, and a specificity of 80.9%. DCA indicated that the model provided a favorable net benefit when the risk threshold exceeded 0.2. The calibration curve demonstrated strong agreement between the predicted and actual values. Furthermore, the Hosmer – Leme show test yielded a P > 0.05, confirming a good model fit (**Figure 4**).

## 4 Discussion

^131^I treatment is widely recognized as a safe and effective treatment for GD, offering distinct advantages such as ease of administration, high safety profile, short treatment duration and low recurrence rate. In recent years, an increasing number of physicians and patients have opted for ^131^I treatment for GD (18). Accurate assessment of thyroid mass is fundamental to determining the appropriate ¹³¹I dosage, which is critical optimizing treatment efficacy and ensuring patient safety.

US imaging is widely recognized for its accuracy in assessing thyroid gland mass within the normal range. However, studies (19) have shown that when thyroid volumes exceed 40 ml, the measurement error of US increases significantly. Certain study (11) evaluated thyroid lobes ranging from 8 to 70 ml and reported an average inaccuracy rate of 16%. In contrast, multiple studies (14, 20) have established CT-based thyroid volumetry as a clinically reliable diagnostic method, indicating excellent agreement with actual thyroid volumes. This technique shows high accuracy in complex thyroid cases, such as multinodular goiters or substernal extensions, as it allows for three-dimensional visualization to enable accurate volume calculations. Consistent with these findings, our study demonstrated that the relative error between CT and the US measurements was 0.19 ± 11.65%. To provide a more intuitive visualization of the relationships and proportional differences between CT and US measurement methods, we employed a Sankey diagram. In this diagram, the width of streamlines, derived from US and CT data nodes, represents the distribution of thyroid mass measurements between the two modalities. Wider streamlines indicate a greater proportion of corresponding mass values within the sample, offering a clearer depiction of the measurement discrepancies between the two techniques (21). Our study demonstrated that in patients with GD, thyroid mass is predominantly distributed in the higher CT cohorts 7 (60–70 g), 8 (70–80 g), and 10 (> 90 g). Furthermore, there is a noticeable tendency for thyroid masses classified under US cohort 8-10 (m > 70 g) to align with CT cohort 10 (m > 90 g), suggesting a systematic underestimation of larger thyroid masses by US. Thyroid mass measurements obtained via CT were consistently higher than those derived from US, highlighting a tendency for US to underestimate larger thyroid mass. This difference is underscored by poor agreement (ICC = 0.179) and significant bias (16.65 ± 15.60 g), indicating that the two methods are not interchangeable in clinical practice. While previous studies have noted this underestimation, a definitive critical threshold had not been established. Using CT as the reference standard, our study identified a significant discrepancy between US and CT measurements when thyroid mass exceeded 20 g. It is demonstrated that for thyroid masses ≤ 20g, US remains a reliable method for assessment, whereas CT calibration is advisable for larger thyroid glands to enhance measurement accuracy.

Multivariate analysis revealed that multiple factors influence treatment outcome, among which disease duration emerged as significant determinant of ^131^I treatment efficacy. Our study demonstrated that patients with longer disease duration tended exhibited poorer prognoses, aligning with findings from previous studies (22, 23). A possible explanation is that prolonged course of GD, often accompanied by extended ATD therapy and recurrent exacerbations, may contribute to autoimmune dysfunction. Persistent TRAb binding to TSH receptors on thyroid cells continuously activates the cAMP signaling pathway, promoting hyperplasia in follicular epithelial cells and lymphoid tissue (24). Such result in the depletion or absence of colloid within thyroid follicles, increasing thyroid stiffness and potentially hindering the therapeutic efficacy of β-radiation in ¹³¹I treatment. Additionally, our comparison of thyroid function before ^131^I treatment revealed that patients with unsuccessful treatment outcomes exhibited elevated FT_4_ levels. Previous studies have reported that GD patients with elevated FT_4_ levels exhibit more pronounced disease severity. This heightened metabolic activity may accelerate the catabolism of internal radiation, thereby diminishing therapeutic responsiveness (25). However, some studies (26, 27) have demonstrated that FT_4_ levels do not significantly influence the success rate of ^131^I treatment. A previous study (28) reported that a higher dose of ¹³¹I was associated with an increased likelihood of therapeutic failure, which aligns with our findings. Conversely, most studies (29, 30) the ¹³¹I dose was higher in the responsive group compared to the non-responsive group. This discrepancy may be attributed to the fact that the maximum initial treatment dose in our study was only 111 MBq (30 mCi), which was considerably lower than that in other studies. Additionally, as thyroid mass increases, US measurement errors become more pronounced. Larger masses often exceed the ultrasound probe’s optimal field of view, causing boundary visualization issues and signal distortions from internal structures like calcifications and substernal extensions (13, 31). These errors lead to underestimating the required ¹³¹I dose, potentially reducing treatment efficacy. Consequently, the administered dose may fall below the therapeutic threshold necessary for achieving a cure, resulting in inconsistent treatment outcomes. Another critical factor influencing the efficacy of ^131^I treatment is thyroid gland uptake. A lower thyroid 24-hour RAIU implies a reduced capacity for iodine retention, leading to decreased ¹³¹I absorption and a shorter effective duration *in vivo*, ultimately compromising therapeutic success. Moreover, individual radiosensitivity may play a key role in determining of the outcome of ^131^I treatment (32). Additionally, although the impact of TRAb on iodine therapy was not prominent in this study, we determined the optimal cut-off value for TRAb to be 38.26 based on the Youden index, which only achieved an AUC value of 0.548. Nevertheless, detection of TRAb changes remains of significant value in the diagnosis of Graves’ disease, as well as in evaluating disease course and recurrence (25).

In the context of the ¹³¹I treatment dosage calculation, thyroid mass serves as a critical determinant in establishing the appropriate therapeutic dosage. Our findings align with the early observations (33), which indicated that larger thyroid mass associated with an increased risk of ¹³¹I treatment failure. This correlation has been further validated by multiple studies. For instance, a retrospective study (34) reported that the 1-year cure rates of the groups with gland weight <30g, 30-60g and >60g were 60.0%, 46.7% and 36.1%, respectively, underscoring the inverse correlation between treatment success and thyroid mass. Similarly, another analysis (35) showed that thyroid mass was the sole determinant of treatment success, with a median mass of 44.6 g in patients who achieved remission. In our study, the identified cut-off value was 35.6 g. The underlying mechanism may involve two key factors. First, an increased thyroid mass can result in inconsistent gland thickness and autoimmune-mediated fibrosis, disrupting the uniform distribution of β-radiation. Second, the incomplete visualization of larger thyroid glands during US may lead to an underestimation of thyroid diameters and overall mass, ultimately causing a miscalculation of the required ¹³¹I dose (36). When the thyroid mass is less than 35.6g, the cure rate of ¹³¹I treatment is relatively high. Given this, a differentiated approach is warranted for patients with GD undergoing initial ^131^I treatment. For those with a normal or mildly enlarged thyroid, the US can serve as the primary assessment modality, effectively minimizing unnecessary radiation exposure from CT. However, in case of larger thyroid glands, CT calibration is recommended to ensure precise ^131^I dose calculation and achieve the intended therapeutic outcome.

This study has several limitations. First, its retrospective design makes it susceptible to selection and statistical biases. Second, the follow - up period was limited to six months, focusing solely on factors affecting the efficacy of a single ¹³¹I treatment. Additionally, the relatively small AUC value for thyroid mass and the moderate accuracy highlights the need for further refinement. Future research should aim to increase sample size, extend the follow – up duration, or explore advanced machine - learning methods to improve predictive accuracy.

For patients with normal or mildly enlarged thyroids, US is sufficient for routine assessment. However, for larger thyroids, CT provides a more precise evaluation, ensuring accurate calculation of the appropriate ^131^I dose. Identifying patients at high - risk clinical factors of non-cure before ¹³¹I treatment is crucial, particularly those with larger thyroid mass. Adjusting the ¹³¹I dose accordingly, with CT calibration when necessary, may enhance the cure rate.

## Data Availability

The raw data supporting the conclusions of this article will be made available by the authors, without undue reservation.
